# DNA motif elucidation using belief propagation

**DOI:** 10.1093/nar/gkt574

**Published:** 2013-06-29

**Authors:** Ka-Chun Wong, Tak-Ming Chan, Chengbin Peng, Yue Li, Zhaolei Zhang

**Affiliations:** ^1^Department of Computer Science, University of Toronto, Toronto, Ontario, Canada, ^2^Terrence Donnelly Centre for Cellular and Biomolecular Research, University of Toronto, Toronto, Ontario, Canada, ^3^Department of Integrative Biology and Physiology, University of California Los Angeles, Los Angeles, CA, USA, ^4^Computer, Electrical and Mathematical Sciences and Engineering Division, King Abdullah University of Science and Technology, Thuwal, Jeddah, KSA, ^5^Banting and Best Department of Medical Research, University of Toronto, Toronto, Ontario, Canada and ^6^Department of Molecular Genetics, University of Toronto, Toronto, Ontario, Canada

## Abstract

Protein-binding microarray (PBM) is a high-throughout platform that can measure the DNA-binding preference of a protein in a comprehensive and unbiased manner. A typical PBM experiment can measure binding signal intensities of a protein to all the possible DNA k-mers (k = 8 ∼10); such comprehensive binding affinity data usually need to be reduced and represented as motif models before they can be further analyzed and applied. Since proteins can often bind to DNA in multiple modes, one of the major challenges is to decompose the comprehensive affinity data into multimodal motif representations. Here, we describe a new algorithm that uses Hidden Markov Models (HMMs) and can derive precise and multimodal motifs using belief propagations. We describe an HMM-based approach using belief propagations (kmerHMM), which accepts and preprocesses PBM probe raw data into median-binding intensities of individual k-mers. The k-mers are ranked and aligned for training an HMM as the underlying motif representation. Multiple motifs are then extracted from the HMM using belief propagations. Comparisons of kmerHMM with other leading methods on several data sets demonstrated its effectiveness and uniqueness. Especially, it achieved the best performance on more than half of the data sets. In addition, the multiple binding modes derived by kmerHMM are biologically meaningful and will be useful in interpreting other genome-wide data such as those generated from ChIP-seq. The executables and source codes are available at the authors’ websites: e.g. http://www.cs.toronto.edu/∼wkc/kmerHMM.

## INTRODUCTION

In human and other higher eukaryotes, gene expression is regulated by the binding of various modulatory transcription factors (TF) onto cis-regulatory DNA elements near genes. Binding of different combinations of TFs may result in a gene being expressed in different tissues or at different developmental stages. To fully understand a gene’s function, it is essential to identify the TFs that regulate the gene and the corresponding TF-binding sites (TFBS). Traditionally, these regulatory sites were determined by labor-intensive experiments such as DNAse footprinting or gel-shift assays. Various computational approaches have been developed to predict TFBS *in silico*, which is an active research area in bioinformatics (1). TFBS are relatively short (10–20 bp) and highly degenerate sequence motifs, which make their effective identification a computationally challenging task. A number of high-throughput experimental technologies were also developed recently to determine protein–DNA-binding affinity.

It is expensive and laborious to experimentally identify TF-TFBS sequence pairs, for example, using DNA footprinting (2) or gel electrophoresis (3). The technology of Chromatin immunoprecipitation (ChIP) followed by microarray or sequencing (4,5) measures the binding occupancy of a particular TF to the nucleotide sequences of co-regulated genes on a genome-wide scale *in vivo* but at low resolution. Further processing is needed to extract precise TFBSs (6). On the other hand, *in vitro* techniques such as protein-binding microarray (PBM) (7), microfluidic affinity analysis (8) and protein microarray assays (9,10) enable us to measure the DNA sequence binding of TFs *in vitro* completely. TRANSFAC is one of the largest databases for regulatory elements including TFs, TFBSs, weight matrices of the TFBSs and regulated genes (11). JASPAR is a comprehensive collection of TF DNA-binding preferences (12). Other annotation databases are also available [e.g. Pfam, UniProbe, ScerTF, FlyTF, YeTFaSCo, hPDI and TFcat (13–19)].

### Background

Numerous studies have been carried out to analyze existing protein–DNA-binding 3D structures comprehensively (20,21) or with focus on specific families [e.g. zinc fingers (22)]. Various properties have been discovered concerning, e.g. bonding and force types, TF conservation and mutation (23) and bending of the DNA (24). Some are already applied to predict binding amino acids on the TF side, e.g. (25,26). Alternatively, researchers have sought for general binding ‘code’ between proteins and DNA, in particular, the one-to-one mapping between amino acids from TFs and nucleotides from TFBSs. Despite many proposed one–one-binding propensity mappings, it has come to a consensus that there is no simple binding ‘code’ (27).

To have a better understanding on protein–DNA-binding motifs, many data mining approaches were proposed and reviewed (28). Researchers use and transfer additional detailed information such as base compositions, structures, thermodynamic properties (29,30) as well as expressions (31), into sophisticated features to fit into certain data mining techniques. These methods usually extract complicated features rather than working on interpretable data directly. Many data-mining techniques, such as neural networks, support vector machines (32) and regressions (28), may generate rules that are difficult to interpret. Furthermore, many data-mining approaches were based on specific protein families or particular data sets. On the other hand, DNA and protein sequences are often the only primary data, which carry important information for protein–DNA-bindings (27,33). Therefore, it is desirable to make use of the existing comprehensive sequence data to discover motif models (34,35).

### Related works

Motif discovery (36) can be categorized into two types: motif scanning and *de novo* motif discovery. (i) Motif scanning is to identify putative TFBSs based on motif knowledge obtained from annotated data (37). (ii) *de novo* motif discovery predicts conserved patterns without knowledge on their appearances, based on mathematical modeling and scoring functions (38,39) from a set of protein/DNA promoter sequences with similar regulatory functions. Although *de novo* motif discovery is successful for well-conserved amino acid domain motifs, the counterpart for DNA remains challenging with less-than-perfect performance on real benchmarks (1,40,36).

To tackle this problem, researchers have used a number of methods to optimize statistical measures, such as Gibbs sampling, expectation maximization, artificial neural network, Markov Chain Monte Carlo, genetic algorithm, maximal information content greedy search approach, simulated annealing, tree data structure, k-mer frequency table, dinucleotide modeling and exhaustive searches (41–55).

It had been pointed out that a fundamental bottleneck in TFBS identification is the lack of quantitative binding affinity data for a large proportion of the TFs. The advancement of new high-throughput technologies such as ChIP-chip, ChIP-seq, protein microarray assays and PBM has made it possible to determine the binding affinity of these TFs (9,10,56). In light of this deluge of quantitative affinity data, traditional approaches that rely on thresholds are no longer adequate. Instead, more robust and probabilistic methods were developed to take into account these quantitative affinity data. Later in the text, we briefly review some of these methods. Seed and Wobble has been proposed as a seed-based approach using rank statistics (7). RankMotif++ was proposed to maximize the log likelihood of their probabilistic model of binding preferences (57). MatrixREDUCE was proposed to perform forward variable selections to minimize the sum of squared deviations (58). MDScan was proposed to combine two search strategies together, namely, word enumeration and position-specific weight matrix updating (6). PREGO was proposed to maximize the Spearman rank correlation between the predicted binding intensities and the measured binding intensities (59). BEEML-PBM was proposed as a regression method to learn an accurate energy model from noisy PBM data (60).

### Problem description

PBM was developed to measure the binding preference of a protein to a complete set of k-mers *in vitro* (7,61). The PBM method has unprecedentedly high resolution and rapid throughput, comparing with the other traditional techniques. It has also been shown to be largely consistent with those generated by *in vivo* genome-wide location analysis (ChIP-chip) (7,61). As a result, researchers have applied this technique onto many TFs, and a large amount of PBM data have been being accumulated and deposited to the UniProbe database (14).

Given a set of DNA sequences, PBM can be used to measure their binding signal intensities for a given DNA-binding protein. Specifically, each probe sequence is associated with a normalized signal intensity value. The higher the normalized signal intensity, the stronger is the binding preference of the DNA-binding protein to the corresponding probe sequence. The actual mathematical relationship between the real binding affinity and the normalized signal intensity is unknown, as it still depends on specific experimental settings (57). Given such data, our goal is to uncover a motif model, which can summarize and represent the DNA-binding preference of the DNA-binding protein. The most common motif model is the Position Weight Matrix (PWM), which assumes independence between adjacent motif positions, justified by the experimental and theoretical statistical mechanical study (62). Although a recent attempt has been made to generalize PWM, the insertion and deletion operations between adjacent nucleotide positions are still challenging (63). In this work, we describe our efforts in developing a hidden Markov models (HMM)-based approach to model the dependence between adjacent nucleotide positions rigorously; we also show that our method (kmerHMM) can also deduce multiple binding modes for a given TF.

## MATERIALS AND METHODS

**Figure 1.**
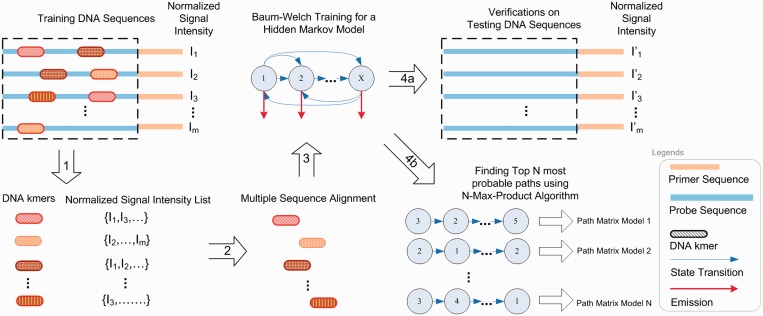
An HMM approach for multimodal motif discovery from PBM data. (1) Positive (bound) k-mers are selected from the training DNA probe sequences (e.g. Array #1). (2) The positive k-mers are aligned using a multiple sequence alignment method. (3) The aligned positive k-mers are input for training an HMM using Baum–Welch training in an unsupervised fashion. (4a) The trained HMM is tested on the testing DNA probe sequences (e.g. Array #2). (4b) The trained HMM can be analyzed and visualized using N-Max-Product algorithm.

Figure 1 illustrates the computational framework that we developed for kmerHMM. For a DNA-binding protein, we are given a set of DNA sequences 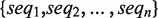 and the corresponding normalized signal intensity values 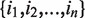 (e.g. Array #1). Following the PBM data analysis convention, we refer to such type of input data set as an array in this manuscript. To extract informative motif data, a sliding window of length *k* is used to scan each DNA sequence (and its reverse complement) to count and record the normalized signal intensity values for each k-mer. Once all the DNA sequences are scanned, a list of normalized signal intensity values is obtained for each k-mer that is present in those DNA sequences. The median of the list is calculated as the median signal intensity *m_x_* for each k-mer *x*. Among those k-mers, some are motif instances (positive k-mers), whereas the others are just background k-mers. To distinguish them, the robust estimate procedures proposed in RankMotif++ (57) is adopted in this work. In other words, we define the positive k-mers to be the k-mers *y* whose median signal intensity 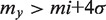 where *mi* and σ are the median and the median absolute deviation (MAD) of the normalized intensities 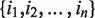 divided by 0.6745 (the MAD of the unit normal distribution), respectively. All the previous numeric settings are set such that the computational condition is consistent with the previous study (57).

After a set of positive k-mers were selected, they are aligned using a multiple sequence alignment method. The aligned k-mers are then input for training an HMM to represent the binding preferences of the DNA-binding protein of interest, using Baum–Welch training algorithm (64). Mathematically, the Baum–Welch training algorithm can be described herein:

**Input:**

A set of aligned k-mers 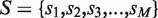 of length *L*. Each k-mer *s_m_* can be represented as 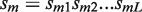 where *s_mp_* is the *p*-th nucleotide of the aligned k-mer *s_m_*:

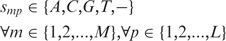

**Output:**

an HMM model θ trained to represent the input aligned k-mers:

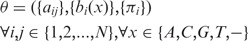

where *a_ij_* is the transition probability from state *i* to state *j*; 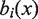 is the emission probability to emit *x* at state *i*; 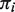 is the initial state probability for state *i*. The mathematical details are available in the Supplementary Data.

After the HMM is trained, each of its hidden state represents a possible nucleotide position in which occurring probabilities of different bases (including gaps) are represented by its emission distribution. The transition probabilities of the HMM encode the indel (insertion/deletion) operations within the motif model implicitly. The advantage of using HMMs over other topologically restricted probabilistic graphical methods is that the graph topology is more flexible so that multimodal motif models can be captured. We subsequently tested the derived HMM model on another set of DNA sequences, which were not used for training (e.g. Array #2). In particular, one may be interested in the ability of the trained HMM to rank the DNA sequences so as to predict which ones are more likely to be the positive probes as well as the correlation between the predicted ranks and measured ranks among the positive probes. On the other hand, N-Max-Product algorithm can be implemented to extract the N most probable paths in its Markov chain, creating multiple motif models in PWM-like forms. Max-product algorithm is a complete generalization of the well-known Viterbi algorithm (65). The major difference is that Viterbi algorithm is given an input sequence and an HMM, whereas max-product algorithm is only given an HMM.

### Parameter settings

The proposed approach was implemented and tested on a previously published PBM data set (7). If the number of positive k-mers is <50, the top 50 k-mers are used to mitigate sampling error. Progressive multiple alignment is adopted; each pairwise alignment is done with the NUC44 scoring matrix (64). After that, pairwise distances between sequences are computed by counting the proportion of sites at which each pair of sequences are similar and different using NUC44 (ignoring gaps). Assuming equal variance and independence of evolutionary distance estimates, the guide tree is calculated by the neighbor-joining method. We have used 50 hidden states for all HMM models trained to achieve rigorous pattern modeling. Such a number of hidden states are chosen based on the empirical performances in a few preliminary runs. Laplace smoothing with 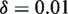 is applied to the emission matrices trained. To be comparable with the previous results, *k* is set to 8, i.e. only 8 mer is considered (57). In particular, we need to control how many steps the Baum–Welch training algorithm executes. In this work, the algorithm terminates when all of the following three quantities become numerically negligible (i.e. <0.1%): (i) the change in the log likelihood that the input sequence is generated by the currently estimated values of the transition and emission matrices; (ii) the change in the norm of the transition matrix, normalized by the size of the matrix; (iii) the change in the norm of the emission matrix, normalized by the size of the matrix. As each HMM is initialized randomly, the training is repeated for 10 times to avoid any suboptimal convergence. Among them, the HMM model with the highest Spearman correlation in the training data is selected as the output HMM model.

We followed the evaluation procedures described in a previous study (57). Specifically, for each DNA-binding protein of interest, we have two array sets of DNA probe sequences, i.e. array #1 and array #2. Each DNA probe sequence on the array is associated with a normalized signal intensity value. The higher the value, the higher is the binding preference of a DNA-binding protein to that DNA sequence. For each DNA-binding protein, the two arrays (data replicates) are alternated for the training and testing purpose. In other words, array #1 is used for training while array #2 is used for testing in the first round, whereas array #2 is used for training while array #1 is used for testing in the second round.

We used two evaluation methods to compare the performance of our method with other previously published methods. The first one is to examine the ability of individual methods to recover and rank the binding preferences of the DNA sequences, whereas the second one is to examine their ability to predict positive DNA sequences among the whole set of testing DNA sequences.

For the first evaluation, Spearman rank correlation coefficients are adopted as the performance metric to compare the true ranking of the binding preferences to the tentative ranking predicted by the different computational methods. To apply kmerHMM to predict sequence rank, we use a sliding window of *L* (i.e. the alignment length in training) to scan each sequence and compute the probability of observing the subsequence within the sliding window using the forward algorithm (65). The maximal probability within each sequence is taken as the quantitative measure for ranking. Mathematically, given a DNA sequence 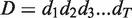, we compute its predicted binding preference *B*(*D*) as:

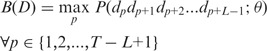

where 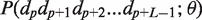 can be computed using the forward algorithm (65), similar to the training procedure described in the previous section.

For the second evaluation, the positive (bound) DNA sequences in each testing data set are defined using the robust estimate in RankMotif++, which was specifically developed for the analyzing raw PBM data (57). The remaining DNA sequences are defined as the negative ones, which accounts for 94.6–99.1% of the testing data set. Given such a two-class classification setting, sensitivities were computed at the 99% specificity level. For kmerHMM, the predicted binding preference 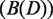 is thresholded to estimate the sensitivities, whereas the other methods used the same settings as described in the previous study (57).

### Max-Product algorithm

In this study, the most probable state transition path 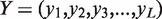 is calculated for each HMM θ trained using the max-product algorithm. Mathematically, Max-Product algorithm can be described herein:

**Input:**

an HMM model θ trained to represent the input aligned k-mers:

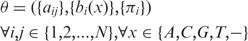

where *a_ij_* is the transition probability from state *i* to state *j*; 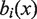 is the emission probability to emit *x* at state *i*; 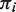 is the initial state probability for state *i*.

**Output:**

Most probable state transition path 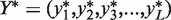 of the input HMM model θ:

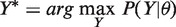

where 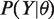 is the probability to have a state transition path *Y* in the input HMM model θ. It can be calculated using a dynamic programming approach. The mathematical details are available in the Supplementary Data.

## RESULTS

### Comparisons

**Figure 2.**
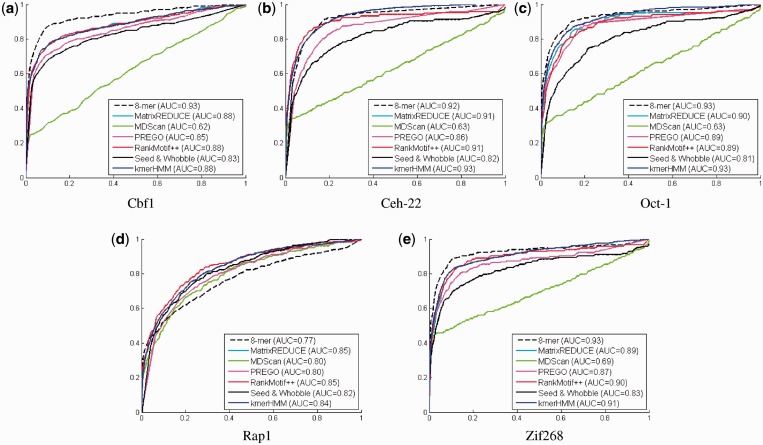
Receiver Operating Characteristic (ROC) curves on array #1. The positive (bound) DNA probe sequences in each data set are defined using the robust estimate in RankMotif++ (57). In other words, we define the positive probes to be the probes *seq_i_*, which normalized signal intensity 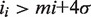 where *mi* and σ are the median and the MAD of all the probe normalized intensities 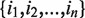 divided by 0.6745 (the MAD of the unit normal distribution), respectively. The remaining ones are defined as the negative ones that accounts for 94.6–99.1% of the data set. Given such a two-class classification setting, the predicted binding preference 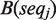 of each probe sequence *seq_j_* is thresholded to estimate the true positive rates at different level of false-positive rates for kmerHMM. The performance values of the other methods are adopted from the RankMotif++ manuscript (57). AUC vstands for the Area Under Curve.

Tables 1 and 2 list the results from our method (kmerHMM). The ROC curves are plotted in Figure 2. From those results, we can observe that kmerHMM performs better than other methods on three datasets (Cbf1, Oct-1 and Zif238). On the two other data sets (Ceh-22 and Rap1), kmerHMM is not the top performer but is close. In the case of Rap1, kmerHMM performed slightly worse than other methods. The consensus binding motif for Rap1 is 13 nt long, which is longer than most of the common TFs. kmeHMM only considers motifs of 8 nt long; therefore, it is at an disadvantage for such cases. Nonetheless, we believe that such a limitation will be alleviated when the PBM technology is improved (i.e. a higher value of k can be used) in the future.

**Table 1.** Spearman rank correlation coefficients

TF	Array	8-mer	MatrixREDUCE	MDScan	PREGO	RankMotif++	Seed and Wobble	kmerHMM
Cbf1	#1	0.647	0.634	0.512	0.61	0.636	0.527	**0.660**
	#2	0.657	0.604	0.496	0.58	0.64	0.49	**0.647**
Ceh-22	#1	0.487	0.373	0.36	0.366	**0.485**	0.304	0.447
	#2	0.408	0.3	0.324	0.278	**0.427**	0.275	0.313
Oct-1	#1	0.327	0.263	0.286	0.281	0.244	**0.315**	0.302
	#2	0.446	0.308	0.264	0.272	0.291	0.213	**0.359**
Rap1	#1	0.238	0.273	0.338	0.261	**0.382**	0.372	0.334
	#2	0.275	0.239	0.254	0.205	**0.359**	0.357	0.270
Zif268	#1	0.421	0.293	0.265	0.292	0.336	0.276	**0.338**
	#2	0.346	0.279	0.246	0.196	0.308	0.25	**0.336**

The rank correlations were computed between the median intensities of the positive probes and the binding preferences predicted by each motif model. The performance values of all the methods except kmerHMM are adopted from Table 1 on the RankMotif++ manuscript (57). The highest values (except the 8-mer gold standard) are highlighted in bold. The 8-mer gold standard is the method in which the maximum of the median binding intensities of the 8-mers on a testing probe (60 bp) is used as the predicted binding preference of the testing probe (60 bp).

**Table 2.** True positive rates at 1% false positive rate

TF	Array	8-mer	MatrixREDUCE	MDScan	PREGO	RankMotif++	Seed and Wobble	kmerHMM
Cbf1	#1	0.515	0.39	0.231	0.362	0.493	0.383	**0.515**
	#2	0.459	0.348	0.202	0.336	0.424	0.284	**0.462**
Ceh-22	#1	0.37	0.26	0.316	0.225	**0.427**	0.254	0.380
	#2	0.257	0.226	0.293	0.2	**0.332**	0.251	0.317
Oct-1	#1	0.474	0.365	0.274	0.339	0.315	0.239	**0.440**
	#2	0.382	0.31	0.213	0.274	0.24	0.202	**0.314**
Rap1	#1	0.257	0.197	0.213	0.197	0.247	0.226	**0.274**
	#2	0.277	0.171	0.32	0.179	**0.325**	0.28	0.243
Zif268	#1	0.449	0.332	0.335	0.328	0.33	0.336	**0.439**
	#2	0.431	0.297	0.314	0.301	0.389	0.313	**0.413**

Given the binding preferences of each method on different data sets, their sensitivities (true-positive rates) were computed at the 99% specificity level (false-positive rate). The performance values of all the methods except kmerHMM are adopted from Table 1 on the RankMotif++ manuscript (57). The highest values (except the 8-mer gold standard) are highlighted in bold. The 8-mer gold standard is the method in which the maximum of the median binding intensities of the 8-mers on a testing probe (60 bp) is used as the predicted binding preference of the testing probe (60 bp).

### Sensitivity analysis

To be comparable with the past results, the positive k-mers are defined as the k-mers *y* whose median signal intensity 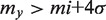 where *mi* and σ are the median and the MAD of the normalized intensities 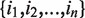 divided by 0.6745 (the MAD of the unit normal distribution), respectively. It is also the threshold to define the positive probes in this study. Nonetheless, the condition may be too stringent; therefore, we have conducted a sensitivity analysis on the condition from 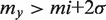 to 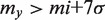 to have a better understanding on kmerHMM. The results are depicted in Supplementary Figures S1 and S2. It can be observed that the area under curve values decrease as the condition becomes more stringent, whereas the spearman rank correlation appears to be fairly stable. Last but not least, the true positive rate also appears to be stable until the condition 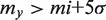, after which the true positive rate drops sharply.

### Positional bias

It has been reported that, in a PBM experiment, the k-mer position on a probe may affect the protein–DNA-binding efficiency (7). Zhao and Stormo proposed a method to take into account the positional bias (60). In kmerHMM, we have adopted the median intensities of the probes containing a k-mer to average out the positional bias. To examine how well such a strategy can deal with the positional bias, we have also implemented and calculated the positional bias coefficients 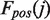 where *j* is the position index (60) for each array and incorporated it into the kmerHMM framework (as shown in Supplementary Figures S3 and S4). It is actually straightforward to integrate it into our kmmHMM framework, as we only need to modify the original *B*(*D*) function to a new function 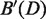 as follows:

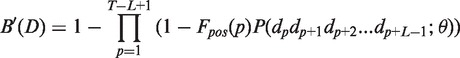

Semantically, the function 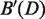 considers all the binding events across the probe and calculate the probability that at least one binding event occurs; each binding event is weighted by the corresponding positional bias coefficient 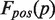.

After the positional bias has been taken into account explicitly, we ran kmerHMM on the data set again. The results are depicted in Supplementary Figure S5. It can be observed that there is a slight improvement on the Cbf1, Ceh-22 and Oct-1 data, whereas slight performance degradation can be seen on the Rap1 and Zif268 data. It is not surprising because Cbf1, Ceh-22 and Oct-1 data have clear and consistent trends in positional bias between Array #1 and #2, which Rap1 and Zif268 do not have (as shown in Supplementary Figures S3 and S4).

### State transition path analysis

#### Max-Product algorithm

In recent years, probabilistic graphical models have been successfully applied to biological problems such as gene clustering and alternative splicing (66–68). In this work, our probabilistic graphical models (i.e. HMM) are learned from the PBM data, which may contain valuable motif information. In particular, we are interested in the most probable path encoded in each HMM trained, as such paths could represent multiple binding modes of a given DNA-binding protein. To solve such a problem, the max-product algorithm (belief propagation) can be used, as it provides us a computationally effective way to avoid the exponential enumerations of the possible state paths using dynamic programming. In addition, its optimality condition has been well studied (69). In this work, we implemented and applied the max-product algorithm to the discrete Markov Chain of each HMM trained. In other words, the most probable state transition path is calculated for each HMM trained. The mathematical formulation can be found in the ‘Materials and Methods’ section.

**Figure 3.**
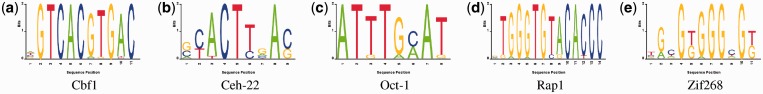
The motif logos mapped and plotted from the most probable state transition paths of the HMMs trained by kmerHMM on Array #1. Those most probable state transition paths are found using the max-product algorithm. The trailing gaps are trimmed.

**Figure 4.**
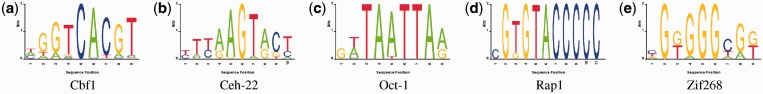
The motif logos mapped and plotted from the most probable state transition paths of the HMMs trained by kmerHMM on Array #2. Those most probable state transition paths are found using the max-product algorithm. The trailing gaps are trimmed.

After the most probable state transition path was calculated for each HMM, we mapped the corresponding emission distribution to each state in the state transition path, resulting in a path model similar to PWM for each HMM. Nonetheless, such a path model is not meant to be equivalent to a PWM, as it only represents the most probable emissions in an HMM, given a fixed path length. The resultant path models are depicted in Figures 3 and 4 for Array #1 and #2, respectively. Comparing them with those past PWM models (57), it can be observed that the most probable paths encoded in kmerHMM are similar to those discovered by the existing PWM-based methods (see Supplementary Figure S6).

#### N-Max-Product algorithm

It has been reported that some DNA-binding proteins could bind to more than one motif models (56). To tackle this, we need to add an additional step to elucidate different motif models from each HMM trained. We herein propose N-Max-Product algorithm to solve the problem. Although the most intuitive solution is to add a sequence clustering step to separate the set of k-mers before the multiple sequence alignment step, such a preprocessing clustering step may lose motif information if the motif models overlap with each other.

To extract the multimodal motif information from the HMMs trained, the N-max-product algorithm (70) is implemented and applied to find the top N most probable state transition paths from the state transition Markov chain in each trained HMM. Using the same method described in the previous section, each state transition path can become an individual path model but, owing to the probabilistic locality, it is likely that those top paths may just be the ones with small variations from the most probable path. Thus, a large value of N needs to be chosen. A clustering step is also needed to summarize them. Specifically, after the top N most probable state transition paths are found, we can map the emission probability distribution to each state in each path. By doing so, we can apply a clustering method to cluster the paths and get a consensus state path model for each cluster.

**Figure 5.**
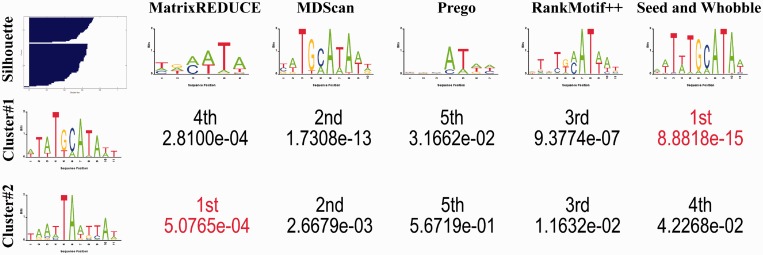
Comparison of PWMs of Oct-1 as predicted by different methods. For the left-most column, the top entry is the silhouette plot for cluster analysis, whereas the other two entries indicate the two centroid path models of the HMMs trained by kmerHMM on the Oct-1 Array #1 data set. The first row shows the sequence logos for the PWMs learned by different methods on the Oct1 Array #1 data set [conducted by Chen *et al.* The figures are edited from (57)]. The remaining numeric entries are the expected values for the pair-wise motif matrix comparisons by STAMP (70). Those two centroids have been confirmed by the independent wet-lab experiments by Verrijzer *et al.* (71).

As an illustrative example, such a procedure was implemented and applied to the Oct-1 Array#1. In the implementation level, we set the value of N such that the (N+1)-th path occurring probability is numerically negligible (i.e. 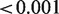). The single-linkage hierarchical clustering was applied to the top N most probable state transition paths to build a cluster dendrogram. To determine the number of clusters, a dendrogram cutoff was chosen such that the mean of the silhouette values was the highest, resulting in two clusters. Their centroids are extracted and depicted with the other sequence logos obtained by the other methods in Figure 5. To quantify their similarities, STAMP was used to calculate the expected value for pair-wise comparisons (71). It can be observed that the first centroid path model is most similar to the one obtained by Seed & Wobble, whereas the second centroid path model is most similar to the one obtained by MatrixREDUCE. Those two motifs have been confirmed by the previous independent wet-lab experiments by Verrijzer *et al.* (72).

We used the two centroid path models to scan the probe sequences in the Oct-1 Array#2 again, following the previous evaluation procedure style. The maximal probability of either path model was taken as the predicted binding preference for each probe sequence. The resultant correlation coefficient between the predicted binding preferences (i.e. ranks) and the measured binding preferences is 0.3264, which is the highest among all the PWM-based methods shown in Table 1. It reflects that kmerHMM can capture multimodal motifs using the HMM modeling and training, explaining why it could perform better than the others in some cases.

We have examined the modes of the state transition paths for the two clusters. Interestingly, we observe that they have different state transition paths, independent of each other. It reflects that HMM modeling is necessary for multimodal motifs, comparing with other modeling in which state transition path topology is restricted to a principal state transition path manually.

### Further evaluation on mouse PBM data

To evaluate kmerHMM further, kmerHMM and RankMotif++ were run and tested on the PBM microarray data provided in the comprehensive mouse data set (56). The results are tabulated in Tables 3 and 4; array #1 is used as training data in Table 3 and array #2 is used as training data in Table 4. Interestingly, it can be observed that kmerHMM can consistently achieve higher true positive rates than RankMotif++, which was specifically designed to analyze PBM data (57).

**Table 3.** Comparisons between kmerHMM and RankMotif++ on the mouse data set (56)

	SR		TPR		AUC			SR		TPR		AUC	
TF	HMM	RM	HMM	RM	HMM	RM	TF	HMM	RM	HMM	RM	HMM	RM
Arid3a	**0.34**	0.13	**0.30**	0.13	**0.91**	0.86	Osr2	**0.20**	0.10	**0.77**	0.07	**0.92**	0.71
Ascl2	**0.42**	0.15	**0.52**	0.07	**0.90**	0.71	Plagl1	0.36	**0.39**	**0.51**	0.27	**0.95**	0.89
Bcl6b	**0.27**	−0.10	**0.17**	0.06	**0.69**	0.63	Rfx3	0.25	**0.30**	**0.29**	0.27	0.86	**0.90**
Bhlhb2	**0.60**	0.46	**0.57**	0.35	**0.92**	0.92	Rfx4	**0.24**	0.15	**0.20**	0.11	**0.80**	0.77
E2F2	**0.43**	0.23	**0.40**	0.23	**0.94**	0.88	Rfxdc2	**0.31**	0.20	**0.42**	0.15	**0.87**	0.79
E2F3	**0.42**	0.20	**0.58**	0.26	**0.98**	0.91	Rxra	**0.38**	0.03	**0.30**	0.02	**0.72**	0.53
Egr1	**0.36**	0.27	**0.57**	0.24	**0.93**	0.84	Sfpi1	0.13	**0.19**	**0.31**	0.14	**0.90**	0.83
Ehf	0.09	**0.24**	**0.79**	0.12	**0.99**	0.77	Sox11	−0.10	**0.12**	**0.25**	0.14	0.71	**0.83**
Elf3	**0.37**	0.23	**0.20**	0.13	0.85	**0.86**	Sox14	0.08	**0.08**	**0.25**	0.11	**0.84**	0.82
Eomes	**0.38**	0.25	0.16	**0.29**	0.78	**0.87**	Sox15	**0.15**	0.02	0.13	**0.25**	0.79	**0.94**
Esrra	**0.43**	0.26	**0.39**	0.21	**0.90**	0.78	Sox17	−0.24	**0.00**	**0.21**	0.10	**0.85**	0.74
Foxa2	**0.29**	−0.01	**0.74**	0.09	**0.97**	0.78	Sox18	**0.20**	0.19	**0.21**	0.14	**0.88**	0.85
Foxj1	0.06	**0.20**	**0.45**	0.13	0.79	**0.84**	Sox21	**0.03**	0.02	**0.15**	0.13	0.76	**0.83**
Foxj3	**0.46**	0.21	**0.35**	0.20	**0.91**	0.86	Sox30	0.06	**0.09**	**0.24**	0.10	0.76	**0.82**
Foxk1	**0.23**	0.02	**0.48**	0.09	**0.92**	0.76	Sox4	0.02	**0.11**	**0.35**	0.13	0.81	**0.82**
Foxl1	**0.47**	0.17	**0.57**	0.17	**0.94**	0.87	Spdef	**0.33**	0.26	**0.30**	0.25	**0.92**	0.88
Gabpa	**0.41**	0.19	**0.33**	0.18	**0.87**	0.86	Srf	**0.28**	0.23	**0.21**	0.01	**0.86**	0.70
Gata3	**0.26**	0.15	**0.31**	0.14	0.87	**0.88**	Sry	**0.15**	−0.02	**0.11**	0.10	**0.90**	0.80
Gata5	**−0.15**	−0.18	**0.44**	0.15	**0.90**	0.73	Tbp	0.16	**0.31**	**0.38**	0.14	0.90	**0.95**
Gata6	0.00	**0.31**	**0.41**	0.14	**0.97**	0.83	Tcf1	**0.14**	0.04	**0.41**	0.09	**0.93**	0.79
Gcm1	**0.35**	0.26	**0.35**	0.14	**0.90**	0.73	Tcf3	**0.36**	−0.16	**0.69**	0.14	**0.93**	0.66
Gm397	**0.34**	0.20	**0.62**	0.13	**0.96**	0.79	Tcf7	**0.26**	0.10	**0.64**	0.10	**0.92**	0.71
Gmeb1	**0.36**	0.12	**0.26**	0.14	**0.88**	0.84	Tcf7l2	**0.50**	0.22	**0.93**	0.07	**1.00**	0.74
Hic1	**0.46**	0.24	**0.41**	0.08	**0.91**	0.75	Tcfap2a	**0.40**	0.31	**0.37**	0.27	**0.91**	0.90
Hnf4a	0.07	**0.21**	**0.50**	0.18	**0.94**	0.83	Tcfap2b	**0.30**	0.25	**0.58**	0.43	**0.98**	0.94
Hoxa3	**0.40**	0.21	**0.37**	0.21	**0.94**	0.86	Tcfap2c	**0.49**	0.28	**0.28**	0.27	0.87	**0.90**
Irf3	**0.23**	0.16	**0.24**	0.14	**0.84**	0.82	Tcfap2e	**0.54**	−0.08	**0.52**	0.07	**0.84**	0.68
Irf4	**0.40**	0.21	**0.20**	0.13	**0.82**	**0.82**	Tcfe2a	**0.69**	0.37	**0.72**	0.28	**0.93**	0.90
Irf5	**0.41**	0.10	**0.29**	0.16	**0.87**	0.83	Zbtb12	**0.32**	−0.10	**0.31**	0.04	**0.71**	0.64
Irf6	**0.39**	0.17	**0.27**	0.11	**0.83**	0.81	Zbtb3	**0.33**	−0.02	**0.65**	0.07	**0.97**	0.72
Isgf3g	**0.29**	0.07	**0.36**	0.11	**0.90**	0.84	Zbtb7b	0.04	0.04	0.39	**0.42**	0.89	**0.97**
Jundm2	**0.50**	0.03	**0.73**	0.06	**0.88**	0.71	Zfp105	**0.27**	0.20	**0.32**	0.19	**0.91**	0.84
Klf7	0.01	**0.08**	**0.77**	0.24	**0.97**	0.82	Zfp128	**0.20**	0.10	**0.74**	0.00	**0.92**	0.74
Mafb	**0.11**	0.07	**0.15**	0.06	**0.71**	0.70	Zfp161	**0.36**	0.29	**0.67**	0.38	**0.98**	0.94
Mafk	**−0.05**	−0.12	**0.25**	0.13	**0.86**	0.81	Zfp281	**0.47**	0.45	**0.70**	0.39	**0.95**	0.88
Max	**0.55**	0.33	**0.50**	0.15	**0.93**	0.85	Zfp410	**0.19**	−0.05	**0.23**	0.04	**0.72**	0.62
Myb	−0.26	**0.13**	**0.50**	0.15	**0.86**	0.79	Zfp691	**0.22**	0.13	**0.76**	0.14	**0.95**	0.83
Mybl1	**0.37**	0.21	**0.37**	0.17	**0.88**	0.82	Zic1	**0.27**	0.18	**0.25**	0.16	**0.86**	0.81
Myf6	**0.29**	0.22	**0.64**	0.03	**0.98**	0.61	Zic2	**0.31**	0.22	**0.21**	0.17	**0.87**	0.82
Nkx3-1	**0.30**	0.18	0.21	**0.21**	**0.87**	0.82	Zic3	**0.29**	0.18	0.14	**0.21**	0.77	**0.85**
Nr2f2	**0.53**	0.28	**0.57**	0.19	**0.93**	0.76	Zscan4	**0.31**	0.18	**0.75**	0.20	**0.96**	0.83
Osr1	**0.13**	−0.02	**0.52**	0.07	**0.79**	0.67							

They have been trained on Array #1 and tested on Array #2 where SR denotes Spearman Rank Correlation, TPR denotes True Positive Rate, AUC denotes Area Under ROC Curve, HMM denotes kmerHMM and RM denotes RankMotif++. The bold values indicate which method (HMM v.s. RM) performs better at a particular test.

**Table 4.** Comparisons between kmerHMM and RankMotif++ on the mouse DNA-binding TF data set (56)

	SR		TPR		AUC			SR		TPR		AUC	
TF	HMM	RM	HMM	RM	HMM	RM	TF	HMM	RM	HMM	RM	HMM	RM
Arid3a	**0.29**	0.17	**0.21**	0.18	**0.94**	0.88	Osr2	**0.40**	0.10	**0.46**	0.07	**0.90**	0.71
Ascl2	**0.27**	0.15	**0.62**	0.07	**0.89**	0.71	Plagl1	**0.49**	0.39	**0.57**	0.27	**0.95**	0.89
Bcl6b	**0.12**	−0.10	**0.10**	0.06	**0.77**	0.63	Rfx3	0.25	**0.30**	**0.36**	0.27	0.90	**0.90**
Bhlhb2	**0.55**	0.46	**0.58**	0.35	0.92	**0.92**	Rfx4	**0.29**	0.15	**0.24**	0.11	**0.85**	0.77
E2F2	**0.36**	0.23	**0.39**	0.23	**0.95**	0.88	Rfxdc2	**0.23**	0.20	**0.43**	0.15	**0.85**	0.79
E2F3	**0.32**	0.20	**0.50**	0.26	**0.96**	0.91	Rxra	**0.29**	0.03	**0.17**	0.02	**0.81**	0.53
Egr1	0.10	**0.27**	**0.31**	0.24	0.84	**0.84**	Sfpi1	0.10	**0.19**	**0.24**	0.14	0.80	**0.83**
Ehf	**0.27**	0.24	**0.32**	0.12	**0.88**	0.77	Sox11	−0.07	**0.12**	**0.28**	0.14	0.74	**0.83**
Elf3	0.15	**0.23**	**0.55**	0.13	**0.97**	0.86	Sox14	−0.15	**0.08**	**0.16**	0.11	0.76	**0.82**
Eomes	0.22	**0.25**	**0.67**	0.29	**0.98**	0.87	Sox15	**0.06**	0.02	0.09	**0.25**	0.75	**0.94**
Esrra	**0.50**	0.26	**0.42**	0.21	**0.94**	0.78	Sox17	−0.05	**0.00**	**0.13**	0.10	0.74	**0.74**
Foxa2	**0.37**	−0.01	**0.52**	0.09	**0.96**	0.78	Sox18	−0.04	**0.19**	0.04	**0.14**	0.74	**0.85**
Foxj1	0.01	**0.20**	**0.22**	0.13	**0.87**	0.84	Sox21	−0.01	**0.02**	**0.14**	0.13	0.74	**0.83**
Foxj3	**0.33**	0.21	**0.32**	0.20	**0.91**	0.86	Sox30	−0.13	**0.09**	**0.18**	0.10	0.74	**0.82**
Foxk1	**0.40**	0.02	**0.33**	0.09	**0.87**	0.76	Sox4	0.20	**0.26**	**0.45**	0.26	0.82	**0.85**
Foxl1	**0.40**	0.17	**0.55**	0.17	**0.94**	0.87	Spdef	0.24	**0.26**	**0.30**	0.25	0.85	**0.88**
Gabpa	**0.38**	0.19	**0.47**	0.18	**0.93**	0.86	Srf	0.11	**0.23**	**0.02**	0.01	**0.73**	0.70
Gata3	**0.19**	0.15	**0.30**	0.14	**0.89**	0.88	Sry	−0.21	**−0.02**	**0.15**	0.10	0.70	**0.80**
Gata5	−0.28	**−0.18**	**0.62**	0.15	**0.91**	0.73	Tbp	−0.12	**0.31**	**0.50**	0.14	0.94	**0.95**
Gata6	**0.33**	0.31	**0.30**	0.14	**0.83**	0.83	Tcf1	−0.08	**0.07**	**0.26**	0.17	0.83	**0.87**
Gcm1	**0.51**	0.26	**0.38**	0.14	**0.90**	0.73	Tcf3	**0.21**	−0.16	**0.38**	0.14	**0.76**	0.66
Gm397	**0.30**	0.20	**0.49**	0.13	**0.86**	0.79	Tcf7	**0.16**	0.10	**0.66**	0.10	**0.89**	0.71
Gmeb1	**0.23**	0.12	**0.17**	0.14	**0.90**	0.84	Tcf7l2	**0.32**	0.22	**0.43**	0.07	**0.92**	0.74
Hic1	**0.42**	0.24	**0.34**	0.08	**0.87**	0.75	Tcfap2a	**0.39**	0.31	**0.41**	0.27	**0.93**	0.90
Hnf4a	0.17	**0.21**	**0.50**	0.18	**0.93**	0.83	Tcfap2b	**0.30**	0.25	**0.54**	0.43	**0.96**	0.94
Hoxa3	**0.48**	0.21	**0.39**	0.21	**0.93**	0.86	Tcfap2c	**0.37**	0.28	**0.45**	0.27	**0.94**	0.90
Irf3	**0.25**	0.16	0.14	**0.14**	0.80	**0.82**	Tcfap2e	**0.42**	−0.08	**0.59**	0.07	**0.91**	0.68
Irf4	**0.38**	0.21	**0.26**	0.13	**0.85**	0.82	Tcfe2a	**0.53**	0.37	**0.61**	0.28	**0.94**	0.90
Irf5	**0.45**	0.10	**0.33**	0.16	**0.88**	0.83	Zbtb12	**0.35**	−0.10	**0.50**	0.04	**0.85**	0.64
Irf6	**0.36**	0.17	**0.39**	0.11	**0.90**	0.81	Zbtb3	**0.27**	−0.02	**0.27**	0.07	**0.83**	0.72
Isgf3g	**0.37**	0.07	**0.45**	0.11	**0.94**	0.84	Zbtb7b	**0.19**	0.04	**0.46**	0.42	0.92	**0.97**
Jundm2	**0.42**	0.03	**0.58**	0.06	**0.96**	0.71	Zfp105	**0.29**	0.20	**0.21**	0.19	**0.85**	0.84
Klf7	**0.17**	0.08	**0.56**	0.24	**0.94**	0.82	Zfp128	−0.20	**0.10**	**0.60**	0.00	**0.80**	0.74
Mafb	−0.03	**0.07**	**0.08**	0.06	0.56	**0.70**	Zfp161	**0.43**	0.29	**0.54**	0.38	**0.97**	0.94
Mafk	**0.27**	−0.12	**0.37**	0.13	**0.87**	0.81	Zfp281	**0.65**	0.45	**0.60**	0.39	**0.91**	0.88
Max	**0.53**	0.33	**0.52**	0.15	**0.95**	0.85	Zfp410	**0.26**	−0.05	**0.25**	0.04	**0.75**	0.62
Myb	**0.21**	0.13	**0.23**	0.15	**0.82**	0.79	Zfp691	**0.39**	0.13	**0.41**	0.14	0.82	**0.83**
Mybl1	**0.27**	0.21	**0.36**	0.17	**0.92**	0.82	Zic1	**0.23**	0.18	**0.17**	0.16	0.80	**0.81**
Myf6	**0.40**	0.22	**0.20**	0.03	**0.74**	0.61	Zic2	**0.24**	0.22	0.17	**0.17**	0.78	**0.82**
Nkx3-1	0.17	**0.18**	**0.65**	0.21	**0.99**	0.82	Zic3	**0.25**	0.18	**0.25**	0.21	**0.88**	0.85
Nr2f2	**0.44**	0.28	**0.43**	0.19	**0.88**	0.76	Zscan4	0.08	**0.18**	**0.80**	0.20	**0.99**	0.83
Osr1	**0.47**	−0.02	**0.27**	0.07	**0.81**	0.67							

They have been trained on Array #2 and tested on Array #1 where SR denotes Spearman Rank Correlation, TPR denotes True Positive Rate, AUC denotes Area Under ROC Curve, HMM denotes kmerHMM and RM denotes RankMotif++. The bold values indicate which method (HMM v.s. RM) performs better at a particular test.

**Figure 6.**
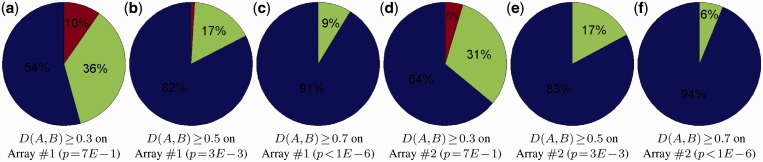
Percentage of multimodal DNA-binding proteins at different distance thresholds 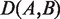 on Array #1 and #2. Blue/Green/Red denote the DNA-binding proteins, which have one/two/three motif matrix model(s), respectively.

Motivated by the good performance of kmmHMM in discovering multimodal binding of DNA-binding proteins, we next used it on all the mouse PBM data to discover how frequently a mouse TF can bind to multiple motifs. Thus, we repeated the previous state transition path analysis using the N-Max-Product algorithm on all the DNA-binding proteins we have studied. After we have removed similar matrix models at different thresholds using 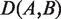 (mathematical details can be found in the Supplementary Data), we have obtained the results depicted in Figure 6. To quantify the statistical significance, we generated two thousand random motif matrix models of width from 5 to 15 uniformly. Nearly 2 million random pair-wise distances have been calculated to estimate the empirical *P*-value distribution for each distance threshold 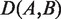 as depicted in Supplementary Figure S7. It can be observed that the distance threshold 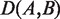 becomes statistically significant at 0.5. At the estimate at 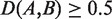 (*P* = 0.003), ∼17% of the mouse DNA-binding proteins we have studied have more than one motif matrix model. Interestingly, it is similar to the estimate in the yeast DNA-binding protein collection, which 26% (39 of 150) proteins have more than one motif matrix model (73).

## DISCUSSION

In this study, we proposed a computational pipeline for PBM motif discovery in which HMMs are trained to model DNA motifs, and Belief Propagation is used to elucidate multiple motif models from each trained HMM. We compared it with other existing methods on benchmark PBM data sets and demonstrated its effectiveness and uniqueness (Tables 1–4 and Figure 5). The novelty of the method lies in two aspects. First, it outperforms the existing method in using HMM to derive an HMM model to represent PBM data. In our knowledge, this is the first instance that HMM is used in representing PBM data. Second, kmerHMM incorporates N-max algorithm and can derive multiple motif matrix models to represent PBM data.

In particular, we implemented a belief propagation method (max-product algorithm) and applied it to the HMMs trained. It can find the most probable state transition paths from the HMMs, representing the DNA-binding preference of the proteins in study. Moreover, the generalized method (N-Max-Product algorithm) has also been implemented and applied. The resultant case study also gave us insights into the multimodal pattern recognition ability of the method proposed. To the best of the authors’ knowledge, this work is the first study incorporating HMMs into the PBM motif discovery problem. In a broader sense, this work is also the first study incorporating max-product algorithms (belief propagations) into the general motif discovery problem explicitly.

The implication of such a study is not limited to motif discovery. From the state transition path analysis, we can observe that HMM training is effective in handling multimodal pattern recognitions, which other modeling methods may not be able to handle. We believe that HMMs should be examined further in other multimodal signal recognition domains. The potential drawback of the proposed approach is that it relies on a sliding window to segment DNA probe sequences into individual k-mers, which may lose the sequence context information. We expect such a limitation will be alleviated when a future improved PBM technology can generate binding affinity for longer probes (i.e. higher k value).

It has been recently intensively debated in the literature that, in light of the availability of high-throughput protein–DNA-binding affinity data, whether there is a need to develop more sophisticated models or simpler position weight matrices are sufficient to capture such binding landscape (60,74). In this work, we demonstrated that kmerHMM can capture multiple binding modes of a DNA-binding protein, for which a single position weight matrix model is unable to do. Nevertheless, we showed that decomposition of the trained HMM into two distinct position weight matrices did show comparable performance to the trained HMM itself on the Oct-1 data set (Spearman rank correlation 0.326 versus 0.359), suggesting that a more sophisticated model such as kmerHMM can achieve better performance, but the overall improvement is likely subtle. However, the strength of the kmerHMM is that it can distinguish distinct binding modes between a DNA-binding protein and its target sequence, which could provide biological insights on the subtlety of the gene regulation. We foresee that a method like kmerHMM is useful in this arena.

## SUPPLEMENTARY DATA

Supplementary Data are available at NAR Online: Supplementary Figures 1–10 and Supplementary Methods.

Supplementary Data

## References

[1] Tompa M, Li N, Bailey TL, Church GM, Moor BD, Eskin E, Favorov AV, Frith MC, Fu Y, Kent WJ (2005). Assessing computational tools for the discovery of transcription factor binding sites. Nat. Biotech..

[2] Galas DJ, Schmitz A (1987). DNAse footprinting: a simple method for the detection of protein-DNA binding specificity. Nucleic Acids Res..

[3] Garner MM, Revzin A (1981). A gel electrophoresis method for quantifying the binding of proteins to specific DNA regions: application to components of the Escherichia coli lactose operon regulatory system. Nucleic Acids Res..

[4] Ren B, Robert F, Wyrick JJ, Aparicio O, Jennings EG, Simon I, Zeitlinger J, Schreiber J, Hannett N, Kanin E (2000). Genome-wide location and function of DNA binding proteins. Science.

[5] Johnson DS, Mortazavi A, Myers RM, Wold B (2007). Genome-wide mapping of in vivo protein-DNA interactions. Science.

[6] Liu XS, Brutlag DL, Liu JS (Aug, 2002). An algorithm for finding protein-DNA binding sites with applications to chromatin-immunoprecipitation microarray experiments. Nat. Biotechnol..

[7] Berger MF, Philippakis AA, Qureshi AM, He FS, Estep PW, Bulyk ML (Nov, 2006). Compact, universal DNA microarrays to comprehensively determine transcription-factor binding site specificities. Nat. Biotechnol..

[8] Fordyce PM, Gerber D, Tran D, Zheng J, Li H, DeRisi JL, Quake SR (2010). De novo identification and biophysical characterization of transcription-factor binding sites with microfluidic affinity analysis. Nat. Biotechnol..

[9] Hu S, Xie Z, Onishi A, Yu X, Jiang L, Lin J, Rho HS, Woodard C, Wang H, Jeong JS (2009). Profiling the human protein-DNA interactome reveals ERK2 as a transcriptional repressor of interferon signaling. Cell.

[10] Ho SW, Jona G, Chen CT, Johnston M, Snyder M (2006). Linking DNA-binding proteins to their recognition sequences by using protein microarrays. Proc. Natl Acad. Sci. USA.

[11] Matys V, Kel-Margoulis OV, Fricke E, Liebich I, Land S, Barre-Dirrie A, Reuter I, Chekmenev D, Krull M, Hornischer K (2006). TRANSFAC and its module TRANSCompel: transcriptional gene regulation in eukaryotes. Nucleic Acids Res..

[12] Portales-Casamar E, Thongjuea S, Kwon AT, Arenillas D, Zhao X, Valen E, Yusuf D, Lenhard B, Wasserman WW, Sandelin A (2010). JASPAR 2010: the greatly expanded open-access database of transcription factor binding profiles. Nucleic Acids Res..

[13] Bateman A, Coin L, Durbin R, Finn RD, Hollich V, GrifRths-Jones S, Khanna A, Marshall M, Moxon S, Sonnhammer ELL (2004). The Pfam protein families database. Nucleic Acids Res..

[14] Robasky K, Bulyk ML (2011). UniPROBE, update 2011: expanded content and search tools in the online database of protein-binding microarray data on protein-DNA interactions. Nucleic Acids Res..

[15] Spivak AT, Stormo GD (2012). ScerTF: a comprehensive database of benchmarked position weight matrices for *Saccharomyces* species. Nucleic Acids Res..

[16] Pfreundt U, James DP, Tweedie S, Wilson D, Teichmann SA, Adryan B (2010). FlyTF: improved annotation and enhanced functionality of the Drosophila transcription factor database. Nucleic Acids Res..

[17] deBoer CG, Hughes TR (2012). YeTFaSCo: a database of evaluated yeast transcription factor sequence specificities. Nucleic Acids Res..

[18] Xie Z, Hu S, Blackshaw S, Zhu H, Qian J (2010). hPDI: a database of experimental human protein-DNA interactions. Bioinformatics.

[19] Fulton DL, Sundararajan S, Badis G, Hughes TR, Wasserman WW, Roach JC, Sladek R (2009). TFCat: the curated catalog of mouse and human transcription factors. Genome Biol..

[20] Luscombe NM, Austin SE, Berman HM, Thornton JM (2000). An overview of the structures of protein-DNA complexes. Genome Biol..

[21] Luscombe NM, Laskowski RA, Thornton JM (2001). Amino acid-base interactions: a three-dimensional analysis of protein-DNA interactions at an atomic level. Nucleic Acids Res..

[22] Krishna SS, Majumdar I, Grishin NV (2003). Structural classification of zinc fingers: survey and summary. Nucleic Acids Res..

[23] Luscombe NM, Thornton JM (2002). Protein-DNA interactions: amino acid conservation and the effects of mutations on binding specificity. J. Mol. Biol..

[24] Jones S, van Heyningen P, Berman HM, Thornton JM (1999). Protein-DNA interactions: a structural analysis. J. Mol. Biol..

[25] Jones S, Shanahan HP, Berman HM, Thornton JM (2003). Using electrostatic potentials to predict DNA-binding sites on DNA-binding proteins. Nucleic Acids Res..

[26] Gunewardena S, Jeavons P, Zhang Z (2006). Enhancing the prediction of transcription factor binding sites by incorporating structural properties and nucleotide covariations. J. Comput. Biol..

[27] Sarai A, Kono H (2005). Protein-DNA recognition patterns and predictions. Annu. Rev. Biophys. Biomol. Struct..

[28] Zhou Q, Liu JS (2008). Extracting sequence features to predict protein-DNA interactions: a comparative study. Nucleic Acids Res..

[29] Ahmad S, Gromiha MM, Sarai A (2004). Analysis and prediction of DNA-binding proteins and their binding residues based on composition, sequence and structural information. Bioinformatics.

[30] Ahmad S, Keskin O, Sarai A, Nussinov R (2008). Protein-DNA interactions: structural, thermodynamic and clustering patterns of conserved residues in DNA-binding proteins. Nucleic Acids Res..

[31] Pham TH, Clemente JC, Satou K, Ho TB (2005). Computational discovery of transcriptional regulatory rules. Bioinformatics.

[32] Ofran Y, Mysore V, Rost B (2007). Prediction of DNA-binding residues from sequence. Bioinformatics.

[33] Wong KC, Peng C, Wong MH, Leung KS (2011). Generalizing and learning protein-DNA binding sequence representations by an evolutionary algorithm. Soft. Comput..

[34] Leung KS, Wong KC, Chan TM, Wong MH, Lee KH, Lau CK, Tsui SK (2010). Discovering protein-DNA binding sequence patterns using association rule mining. Nucleic Acids Res..

[35] Chan TM, Wong KC, Lee KH, Wong MH, Lau CK, Tsui SK, Leung KS (2011). Discovering approximate-associated sequence patterns for protein-DNA interactions. Bioinformatics.

[36] MacIsaac KD, Fraenkel E (2006). Practical strategies for discovering regulatory DNA sequence motifs. PLoS Comput. Biol..

[37] Kel AE, Goessling E, Reuter I, Cheremushkin E, Kel-Margoulis OV, Wingender E (2003). MATCH: a tool for searching transcription factor binding sites in DNA sequences. Nucleic Acids Res..

[38] Stormo GD (1988). Computer methods for analyzing sequence recognition of nucleic acids. Annu. Rev. BioChem..

[39] Jensen ST, Liu XS, Zhou Q, Liu JS (2004). Computational discovery of gene regulatory binding motifs: a Bayesian perspective. Stat. Sci..

[40] Sandve GK, Abul O, Walseng V, Drablos F (2007). Improved benchmarks for computational motif discovery. BMC Bioinformatics.

[41] Hughes JD, Estep PW, Tavazoie S, Church GM (2000). Computational identification of cis-regulatory elements associated with groups of functionally related genes in *Saccharomyces cerevisiae*. J. Mol. Biol..

[42] Thijs G, Lescot M, Marchal K, Rombauts S, DeMoor B, Rouze P, Moreau Y (2001). A higher-order background model improves the detection of promoter regulatory elements by Gibbs sampling. Bioinformatics.

[43] Ao W, Gaudet J, Kent WJ, Muttumu S, Mango SE (2004). Environmentally induced foregut remodeling by PHA-4/FoxA and DAF-12/NHR. Science.

[44] Bailey TL, Elkan C (1995). The value of prior knowledge in discovering motifs with MEME. Proc. Int. Conf. Intell. Syst. Mol. Biol..

[45] Workman CT, Stormo GD (2000). ANN-Spec: a method for discovering transcription factor binding sites with improved specificity. Pac. Symp. Biocomput..

[46] Favorov AV, Gelfand MS, Gerasimova AV, Ravcheev DA, Mironov AA, Makeev VJ (2005). A Gibbs sampler for identification of symmetrically structured, spaced DNA motifs with improved estimation of the signal length. Bioinformatics.

[47] Chan TM, Leung KS, Lee KH (2008). TFBS identification based on genetic algorithm with combined representations and adaptive post-processing. Bioinformatics.

[48] Hertz GZ, Stormo GD (1999). Identifying DNA and protein patterns with statistically significant alignments of multiple sequences. Bioinformatics.

[49] Frith MC, Hansen U, Spouge JL, Weng Z (2004). Finding functional sequence elements by multiple local alignment. Nucleic Acids Res..

[50] Eskin E, Pevzner PA (2002). Finding composite regulatory patterns in DNA sequences. Bioinformatics.

[51] van Helden J, Andre B, Collado-Vides J (1998). Extracting regulatory sites from the upstream region of yeast genes by computational analysis of oligonucleotide frequencies. J. Mol. Biol..

[52] Gunewardena S, Zhang Z (2008). A hybrid model for robust detection of transcription factor binding sites. Bioinformatics.

[53] Régnier M, Denise A (2004). Rare events and conditional events on random strings. Discrete Math..

[54] Pavesi G, Mereghetti P, Mauri G, Pesole G (2004). Weeder Web: discovery of transcription factor binding sites in a set of sequences from co-regulated genes. Nucleic Acids Res..

[55] Sinha S, Tompa M (2003). YMF: A program for discovery of novel transcription factor binding sites by statistical overrepresentation. Nucleic Acids Res..

[56] Badis G, Berger MF, Philippakis AA, Talukder S, Gehrke AR, Jaeger SA, Chan T, Metzler G, Vedenko A, Chen X (2009). Diversity and complexity in DNA recognition by transcription factors. Science.

[57] Chen X, Hughes TR, Morris Q (2007). RankMotif++: a motif-search algorithm that accounts for relative ranks of K-mers in binding transcription factors. Bioinformatics.

[58] Foat BC, Houshmandi SS, Olivas WM, Bussemaker HJ (2005). Profiling condition-specific, genome-wide regulation of mRNA stability in yeast. Proc. Natl Acad. Sci. USA.

[59] Tanay A (2006). Extensive low-affinity transcriptional interactions in the yeast genome. Genome Res..

[60] Zhao Y, Stormo GD (2011). Quantitative analysis demonstrates most transcription factors require only simple models of specificity. Nat. Biotechnol..

[61] Weirauch MT, Cote A, Norel R, Annala M, Zhao Y, Riley TR, Saez-Rodriguez J, Cokelaer T, Vedenko A, Talukder S (2013). Evaluation of methods for modeling transcription factor sequence specificity. Nat. Biotechnol..

[62] Berg OG, von Hippel PH (1987). Selection of DNA binding sites by regulatory proteins. Statistical-mechanical theory and application to operators and promoters. J. Mol. Biol..

[63] Stormo GD (2011). Maximally efficient modeling of DNA sequence motifs at all levels of complexity. Genetics.

[64] Durbin R, Eddy SR, Krogh A, Mitchison G (1998). Biological Sequence Analysis: Probabilistic Models of Proteins and Nucleic Acids.

[65] Rabiner LR (1990). Readings in Speech Recognition. Chapter A Tutorial on Hidden Markov Models and Selected Applications in Speech Recognition.

[66] Frey BJ, Mohammad N, Morris QD, Zhang W, Robinson MD, Mnaimneh S, Chang R, Pan Q, Sat E, Rossant J (2005). Genome-wide analysis of mouse transcripts using exon microarrays and factor graphs. Nat. Genet..

[67] Frey BJ, Dueck D (2007). Clustering by passing messages between data points. Science.

[68] Barash Y, Calarco JA, Gao W, Pan Q, Wang X, Shai O, Blencowe BJ, Frey BJ (2010). Deciphering the splicing code. Nature.

[69] Weiss Y, Freeman WT (2001). On the optimality of solutions of the max-product belief-propagation algorithm in arbitrary graphs. IEEE Trans. Inf. Theory..

[70] Barber D (2011). Bayesian Reasoning and Machine Learning.

[71] Mahony S, Benos PV (2007). STAMP: a web tool for exploring DNA-binding motif similarities. Nucleic Acids Res..

[72] Verrijzer CP, Alkema MJ, van Weperen WW, VanLeeuwen HC, Strating MJ, vander Vliet PC (1992). The DNA binding specificity of the bipartite POU domain and its subdomains. EMBO J..

[73] Gordan R, Murphy KF, McCord RP, Zhu C, Vedenko A, Bulyk ML (2011). Curated collection of yeast transcription factor DNA binding specificity data reveals novel structural and gene regulatory insights. Genome Biol..

[74] Morris Q, Bulyk ML, Hughes TR (2011). Jury remains out on simple models of transcription factor specificity. Nat. Biotechnol..

