# Supplementary material for: DNA motif elucidation using belief propagation
Source: Nucleic Acids Res. 2013 Jun 29;41(16):e153. doi: 10.1093/nar/gkt574 (PMC3763557; doi:10.1093/nar/gkt574)
Supplement: Supplementary Data [file supp_41_16_e153__index.html]

DNA motif elucidation using belief propagation — DNA motif elucidation using belief propagation — Supplementary Data 

# DNA motif elucidation using belief propagation

## 

files

**Files in this Data Supplement:**

- Supplementary Data - pdf file
