# Supplementary material for: DNA motif elucidation using belief propagation
Source: Nucleic Acids Res. 2013 Jun 29;41(16):e153. doi: 10.1093/nar/gkt574 (PMC3763557; doi:10.1093/nar/gkt574)
Supplement: Supplementary Data [file supp_gkt574_nar-00905-met-n-2013-File002.pdf]

Published online *Nucleic Acids Research, , Vol. , No.* **1–S12**
doi:10.1093/nar/gkn000

Supplementary Data for The Manuscript titled "DNA Motif Elucidation using Belief Propagation"

Ka-Chun Wong [1,2], Tak-Ming Chan [3], Chengbin Peng [4], Yue Li [1,2], Zhaolei Zhang [1,2,5,6] *

[1]Department of Computer Science, University of Toronto, Toronto, Ontario, Canada and [2]Terrence Donnelly Centre for Cellular and Biomolecular Research, University of Toronto, Toronto, Ontario, Canada and [3]Department of Integrative Biology and Physiology, University of California Los Angeles, Los Angeles, California, U.S.A. and [4]CEMSE Division, King Abdullah University of Science and Technology, Thuwal, Jeddah, K.S.A. and [5]Banting and Best Department of Medical Research, University of Toronto, Toronto, Ontario, Canada and [6]Department of Molecular Genetics, University of Toronto, Toronto, Ontario, Canada

*To whom correspondence should be addressed. Tel: +416 946 0924; Fax: +416 946 0924; Email: zhaolei.zhang@utoronto.ca

© The Author(s)
This is an Open Access article distributed under the terms of the Creative Commons Attribution Non-Commercial License (http://creativecommons.org/licenses/by-nc/2.0/uk/) which permits unrestricted non-commercial use, distribution, and reproduction in any medium, provided the original work is properly cited.

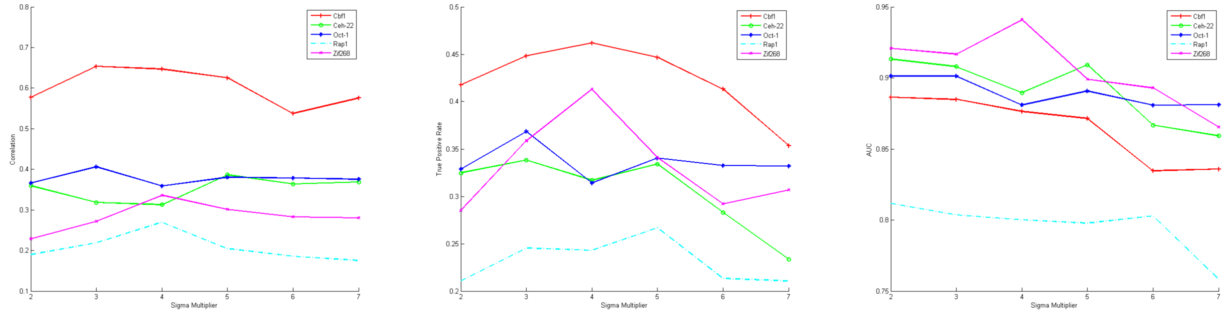

(a) Spearman Correlation (b) True Positive Rate (c) Area Under Curve

Figure S1. Performance curves of kmerHMM trained on Array $\#1$ and tested on Array $\#2$. The positive k-mer and probe condition varies from $m_y > mi + 2\sigma$ to $m_y > mi + 7\sigma$ along the horizontal axis, while the vertical axis indicates performance values.

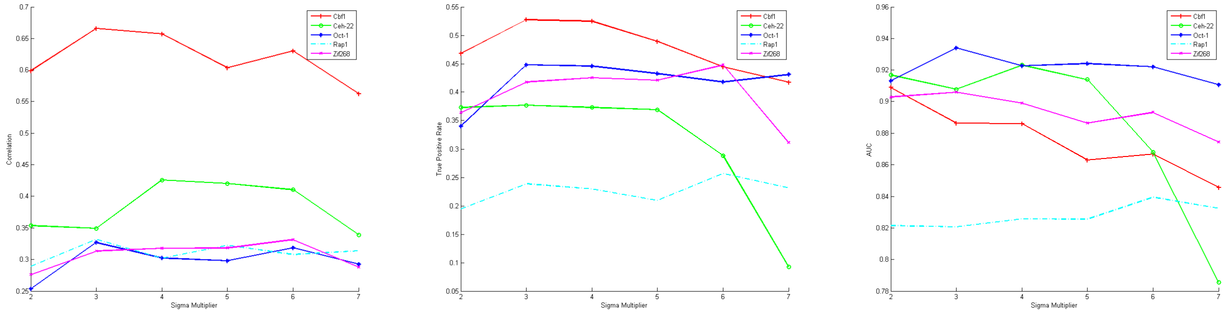

(a) Spearman Correlation (b) True Positive Rate (c) Area Under Curve

Figure S2. Performance curves of kmerHMM trained on Array $\#2$ and tested on Array $\#1$. The positive k-mer and probe condition varies from $m_y > mi + 2\sigma$ to $m_y > mi + 7\sigma$ along the horizontal axis, while the vertical axis indicates performance values.

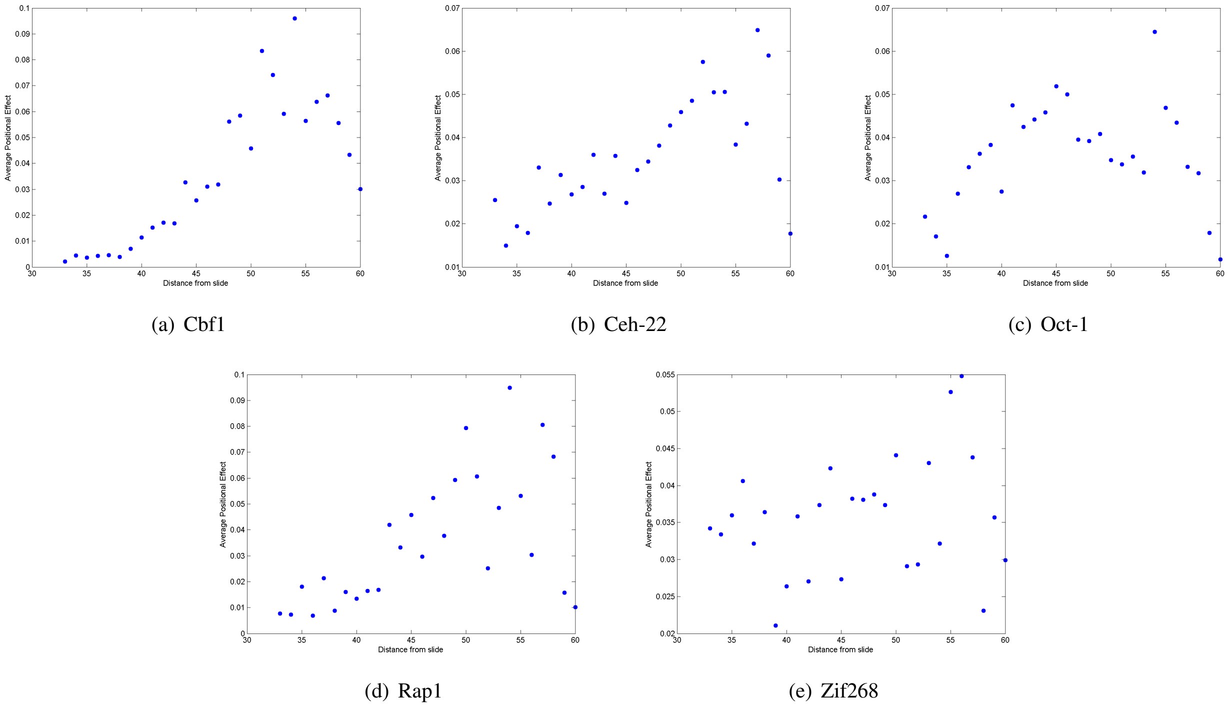

Figure S3. Positional Bias Coefficient F_{pos} estimated from Array #1. Its estimation method can be found in (60).

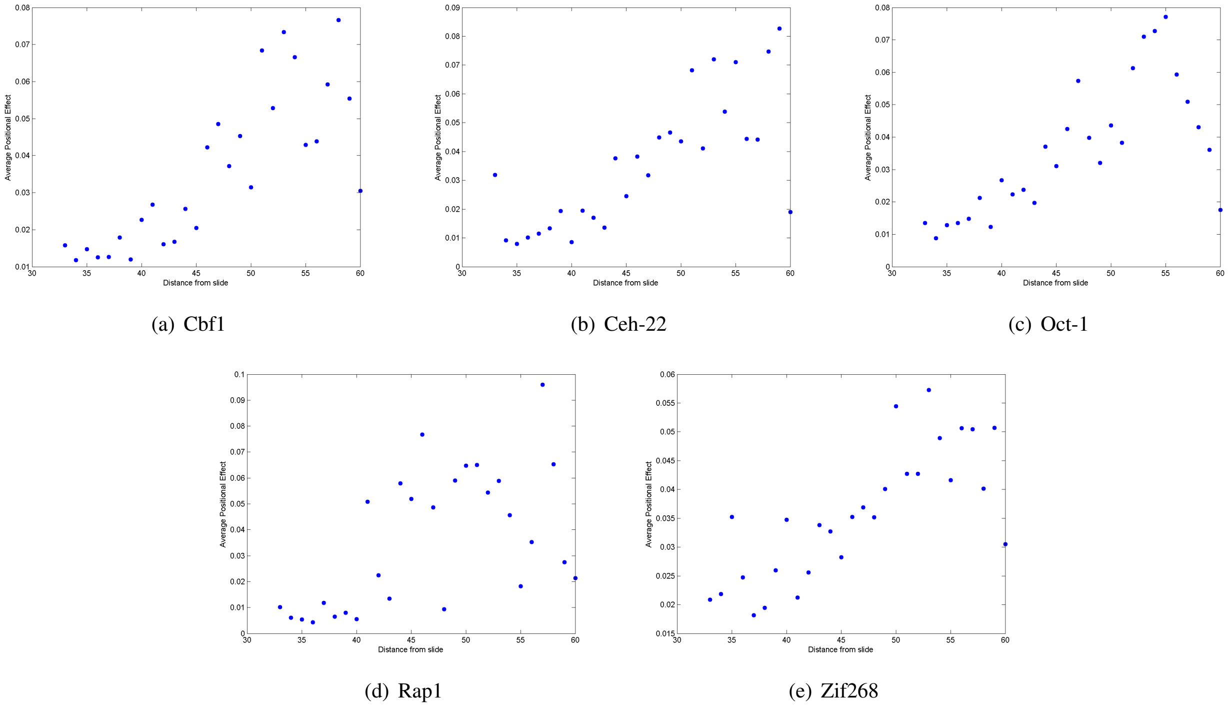

Figure S4. Positional Bias Coefficient F_{pos} estimated from Array #2. Its estimation method can be found in (60).

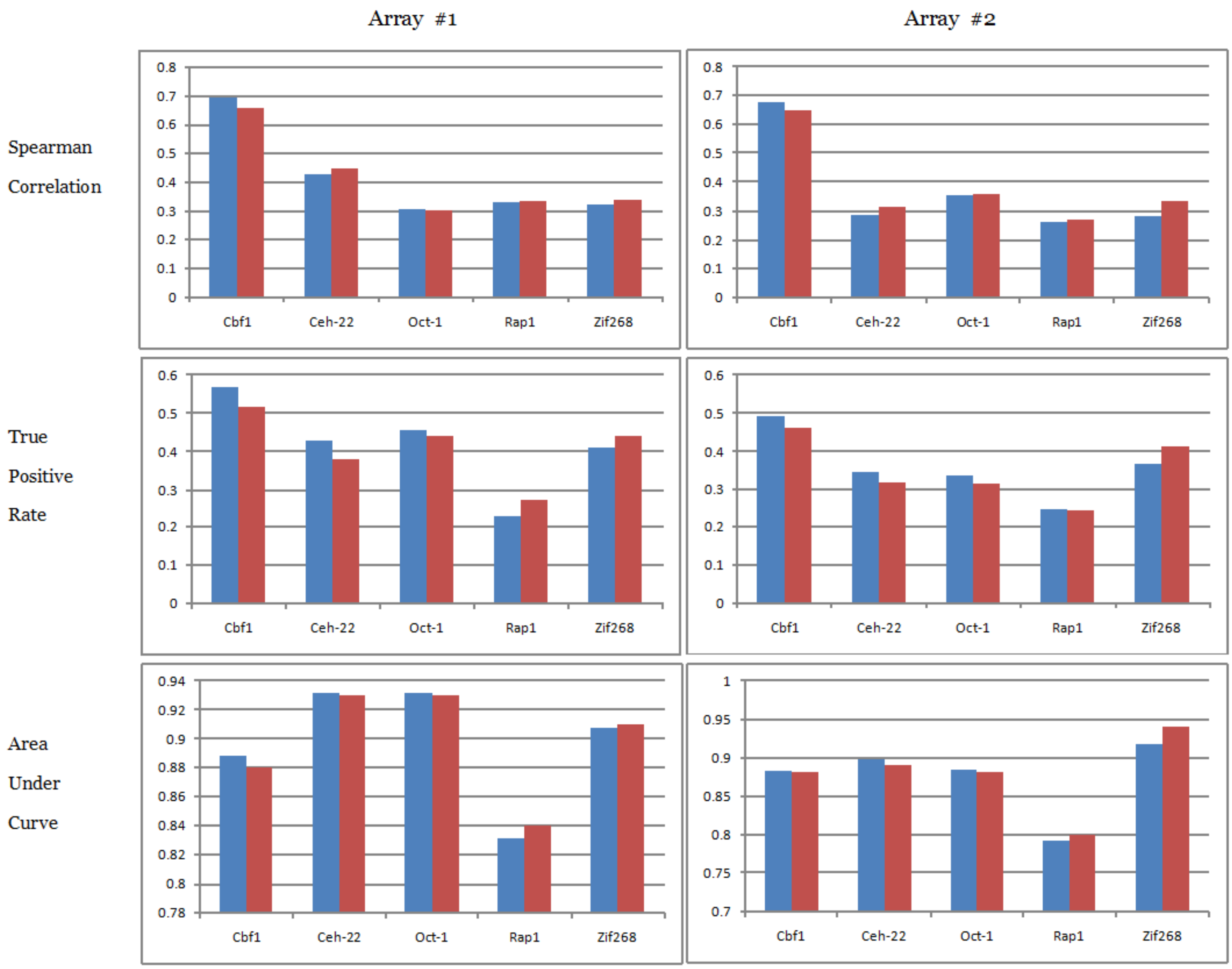

Figure S5. Performance Difference for kmerHMM with (in blue color) and without (in red color) positional bias correction.

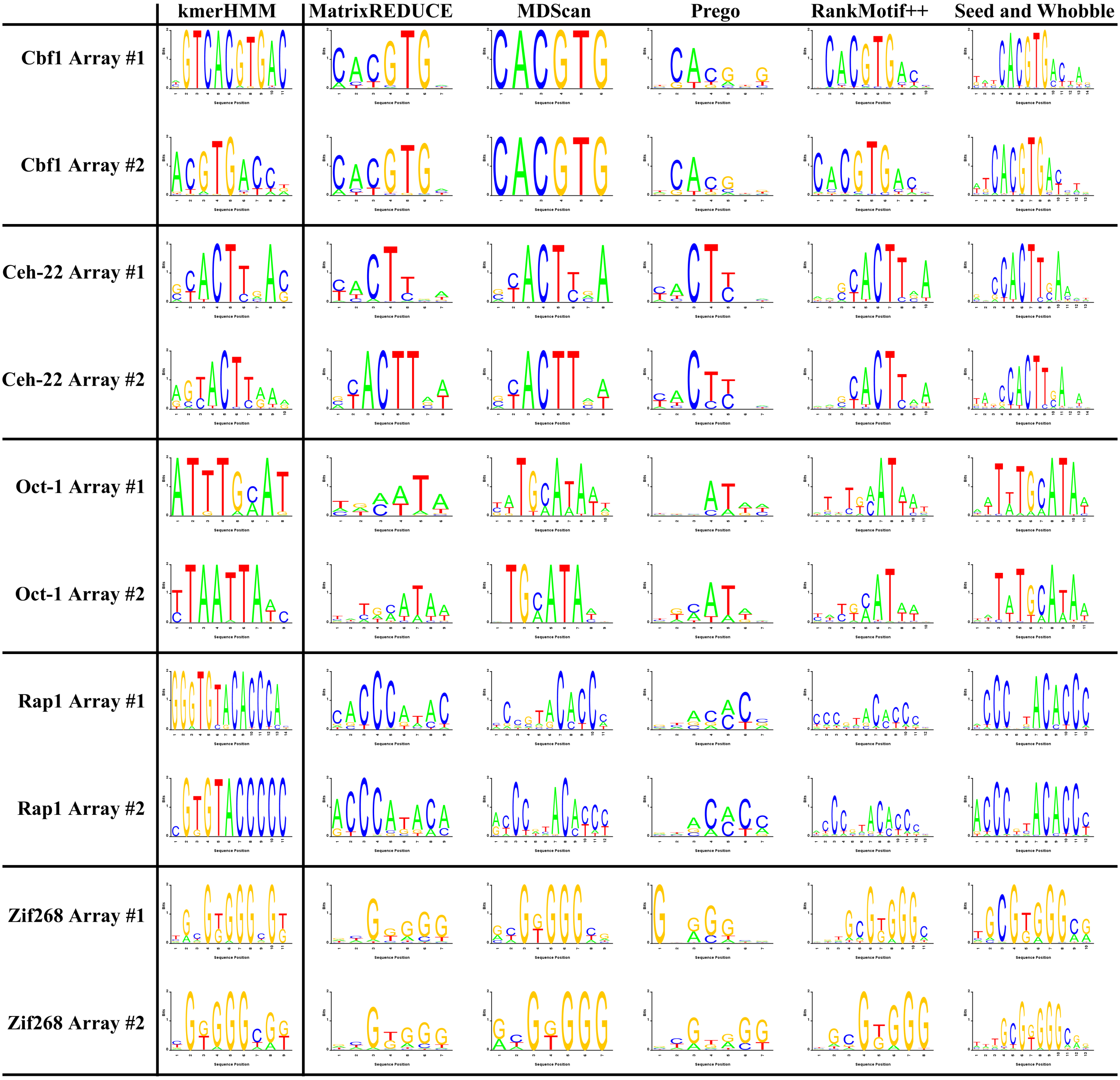

Figure S6. Motif Comparison. The motif logos mapped and plotted from the most probable state transition paths of the HMMs trained by kmerHMM are found using the max-product algorithm. It can be seen that the most probable state transition paths of kmerHMM encode the patterns similar to the motif matrices found by other methods.

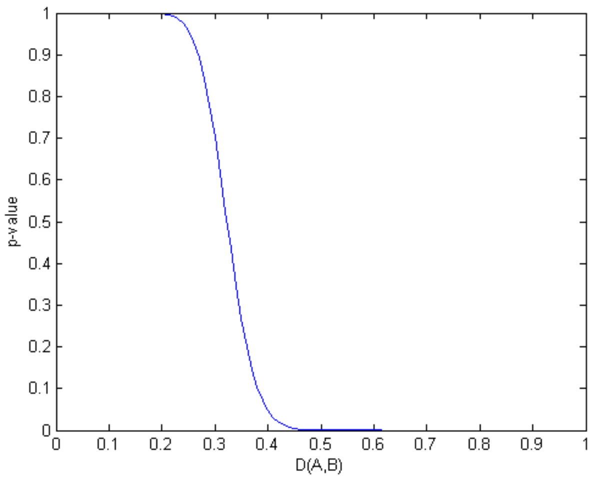

Figure S7. Empirical p-values estimated from about 2 million random pair-wise distances.

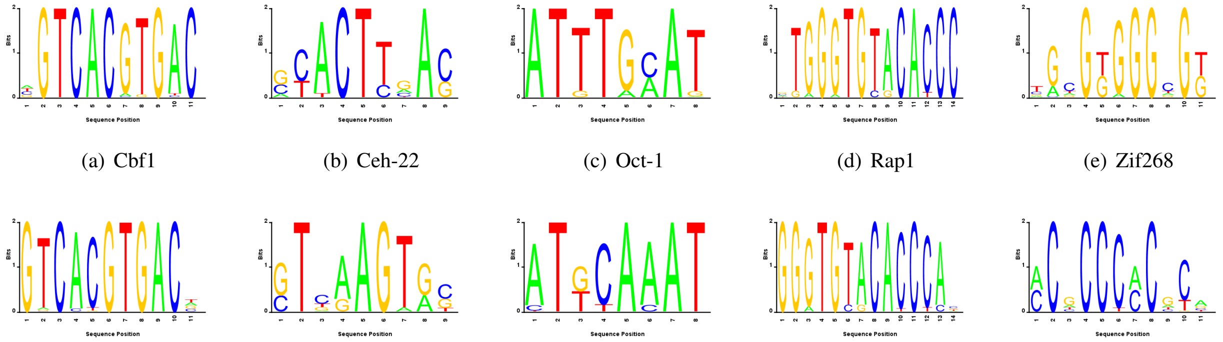

(a) Cbf1 (b) Ceh-22 (c) Oct-1 (d) Rap1 (e) Zif268

(f) Cbf1(RC) (g) Ceh-22(RC) (h) Oct-1(RC) (i) Rap1(RC) (j) Zif268(RC)

Figure S8. The motif logos mapped and plotted from the most probable state transition paths of the HMMs trained by kmerHMM on Array #1 are found using the max-product algorithm. The trailing gaps are trimmed. RC stands for Reverse Complement.

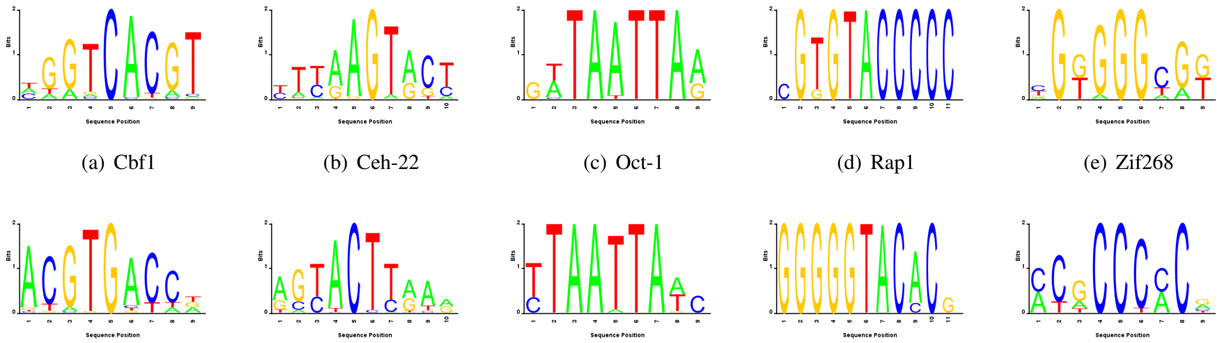

(a) Cbf1 (b) Ceh-22 (c) Oct-1 (d) Rap1 (e) Zif268

(f) Cbf1(RC) (g) Ceh-22(RC) (h) Oct-1(RC) (i) Rap1(RC) (j) Zif268(RC)

Figure S9. The motif logos mapped and plotted from the most probable state transition paths of the HMMs trained by kmerHMM on Array #2 are found using the max-product algorithm. The trailing gaps are trimmed. RC stands for Reverse Complement.

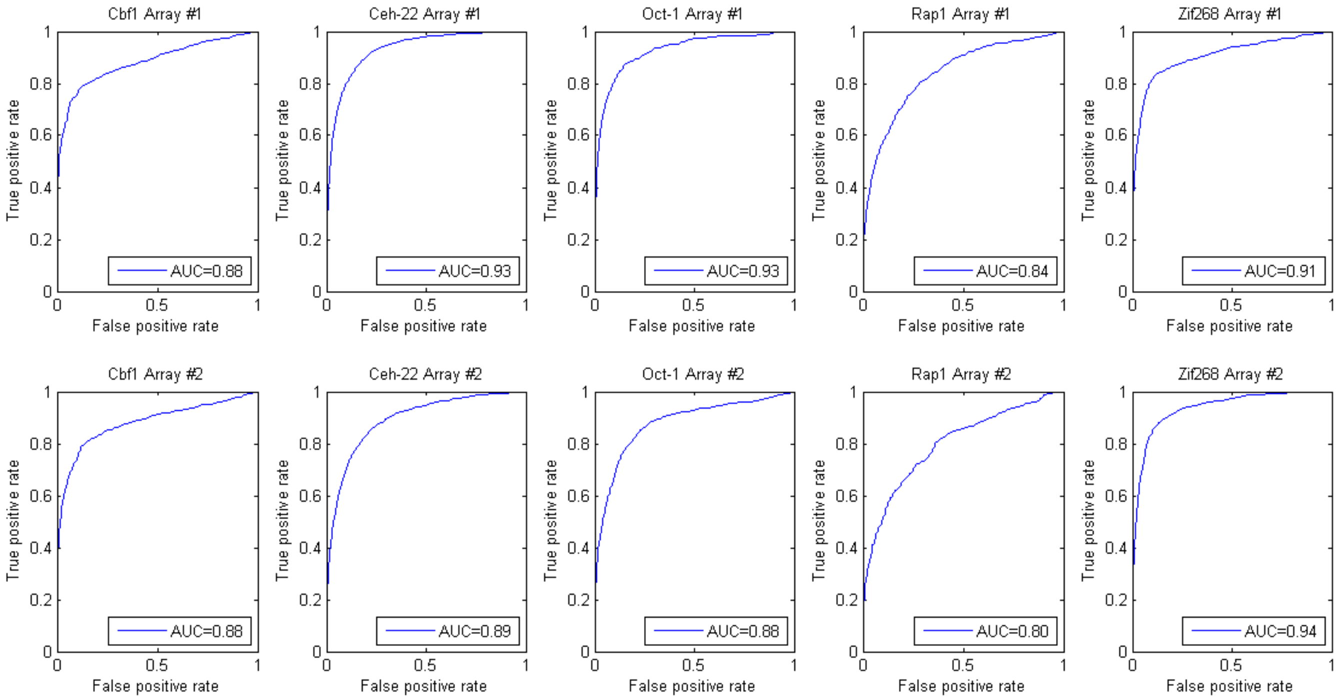

Figure S10. Receiver Operating Characteristic (ROC) curves for kmerHMM trained on Array $\#1$ and $\#2$ and tested on Array $\#2$ and $\#1$ respectively. The positive (bound) DNA probe sequences in each dataset are defined using the robust estimate in RankMotif++ (57). In other words, we define the positive probes to be the probes seq_i which normalized signal intensity $i_i > mi + 4\sigma$ where mi and σ are the median and the median absolute deviation (MAD) of all the probe normalized intensities $\{i_1, i_2, ..., i_n\}$ divided by 0.6745 (the MAD of the unit normal distribution) respectively. The remaining ones are defined as the negative ones which accounts for 94.6% to 99.1% of the dataset. Given such a two-class classification setting, the predicted binding preference $B(seq_j)$ of each probe sequence seq_j is thresholded to estimate the true positive rates at different level of false positive rates where AUC stands for the Area Under Curve.

BAUM WELCH ALGORITHM

After a set of positive k-mers were selected, they are aligned using a multiple sequence alignment method. The aligned k-mers are then input for training a HMM to represent the binding preferences of the DNA-binding protein of interest, using Baum Welch training algorithm (65).

Mathematically, the Baum Welch training algorithm can be described herein:

Input: A set of aligned k-mers $S=\{s_1,s_2,s_3,...,s_M\}$ of length L. Each k-mer s_m can be represented as $s_m=s_{m1}s_{m2}...s_{mL}$ where s_{mp} is the p-th nucleotide of the aligned k-mer s_m:

$$s_{mp}\in\{A,C,G,T,-\}$$

$$\forall m\in\{1,2,...,M\},\forall p\in\{1,2,...,L\}$$

Output: A HMM model θ trained to represent the aligned k-mers:

$$\theta=(\{a_{ij}\},\{b_i(x)\},\{\pi_i\})$$

$$\forall i,j\in\{1,2,...,N\},\forall x\in\{A,C,G,T,-\}$$

where a_{ij} is the transition probability from state i to state j; $b_i(x)$ is the emission probability to emit x at state i; π_i is the initial state probability for state i.

As Baum Welch training is an Expectation Maximization (EM) algorithm, we randomly initialize those HMM model parameters at the beginning θ_0 and iteratively refine them in each iteration.

In the expectation step (**E-step**) of the l-th iteration, we calculate the expected values of being in state i based on the current parameter estimates θ_l. Specifically, we calculate:

$$\gamma_p^m(i)=\frac{\alpha_p^m(i)\beta_p^m(i)}{P(s_m;\theta_l)}$$

where $\gamma_p^m(i)$ is the expected probability of being in state i at the p-th nucleotide position for the aligned k-mer s_m; $\alpha_p^m(i)$ and $\beta_p^m(i)$ are the forward and backward probability of the aligned k-mer s_m to be in state i at the p-th nucleotide position as calculated by the dynamic programming approach (65). $P(s_m;\theta_l)$ is the probability of observing s_m given the existing HMM model parameter θ_l which can be calculated as $P(s_m;\theta_l)=\sum_{i=1}^N\alpha_p^m(i)\beta_p^m(i)$. In addition, we also calculate the expected values of state transitions from state i to state j:

$$\zeta_p^m(i,j)=\frac{\alpha_p^m(i)a_{ij}b_j(s_{mp})\beta_{p+1}^m(j)}{P(s_m;\theta_l)}$$

where $\zeta_p^m(i,j)$ is the expected probability of transiting from state i at the nucleotide position p to state j at the nucleotide position $p+1$ for the aligned k-mer s_m given the current parameter estimates θ_l.

In the maximization step (**M-step**) of the l-th iteration, those model parameters are refined to be the maximal likelihood estimates for those expected values:

$$\pi_i'=\sum_{m=1}^M\gamma_1^m(i)$$

$$a_{ij}'=\frac{\sum_{m=1}^M\sum_{p=1}^{L-1}\zeta_p^m(i,j)}{\sum_{m=1}^M\sum_{p=1}^{L-1}\gamma_p^m(i)}$$

$$b_i(x)'=\frac{\sum_{m=1}^M\sum_{p=1}^{L}\gamma_p^m(i)[s_{mp}=x]}{\sum_{m=1}^M\sum_{p=1}^{L}\gamma_p^m(i)}$$

$$\theta_{l+1}=(\{a_{ij}'\},\{b_i(x)'\},\{\pi_i'\})$$

The new HMM model parameters θ_{l+1} is used in the next iteration. We repeat the E-step and M-step alternatively until the HMM model parameters are not changed anymore. In other words, the difference between θ_l and θ_{l+1} converges to a numerically negligible value at which a local optimum is found.

MAX-PRODUCT ALGORITHM

In this study, the most probable state transition path $Y = (y_1, y_2, y_3, ..., y_L)$ is calculated for each HMM θ trained using the max-product algorithm. Mathematically: Max-Product algorithm can be described herein:

Input: A HMM model θ trained to represent the input aligned k-mers:

$$\theta = (\{a_{ij}\}, \{b_i(x)\}, \{\pi_i\})$$
$$\forall i, j \in \{1, 2, ..., N\}, \forall x \in \{A, C, G, T, -\}$$

where a_{ij} is the transition probability from state i to state j; $b_i(x)$ is the emission probability to emit x at state i; π_i is the initial state probability for state i.

Output: Most probable state transition path $Y^* = (y_1^*, y_2^*, y_3^*, ..., y_L^*)$ in the input HMM model θ:

$$Y^* = \arg\max_Y P(Y|\theta)$$

which can be calculated using a dynamic programming approach. If we expand $P(Y|\theta)$ to the classic likelihood function $P(Y, O|\theta)$ of a HMM, we can further simplify it:

$$\arg\max_Y \sum_O P(Y, O|\theta)$$
$$\Rightarrow \arg\max_Y \sum_O P(y_1)P(y_2|y_1)P(y_3|y_2)...P(y_L|y_{L-1})$$
$$P(o_1|y_1)P(o_2|y_2)...P(o_L|y_L)$$
$$\Rightarrow \arg\max_Y P(y_1)P(y_2|y_1)P(y_3|y_2)...P(y_L|y_{L-1})$$
$$\sum_{o_1} P(o_1|y_1) \sum_{o_2} P(o_2|y_2) ... \sum_{o_L} P(o_L|y_L)$$
$$\Rightarrow \arg\max_Y P(y_1)P(y_2|y_1)P(y_3|y_2)...P(y_L|y_{L-1})$$

It is reduced to finding the most probable state transition path for the discrete Markov chain of the HMM θ. It can be computed using a dynamic programming approach (69) as follows:

$$\arg\max_{y_1} P(y_1) \arg\max_{y_2} P(y_2|y_1) \arg\max_{y_3} P(y_3|y_2)...$$
$$\arg\max_{y_{L-1}} P(y_{L-1}|y_{L-2}) \arg\max_{y_L} P(y_L|y_{L-1})$$
$$\Rightarrow \arg\max_{y_1}(\pi_{y_1}) \arg\max_{y_2}(a_{y_1 y_2}) \arg\max_{y_3}(a_{y_2 y_3})...$$
$$\arg\max_{y_{L-1}}(a_{y_{L-2} y_{L-1}}) \arg\max_{y_L}(a_{y_{L-1} y_L})$$
$$\Rightarrow \arg\max_{y_1}(\pi_{y_1}) \arg\max_{y_2}(a_{y_1 y_2}) \arg\max_{y_3}(a_{y_2 y_3})...$$
$$\arg\max_{y_{L-1}}(a_{y_{L-2} y_{L-1}} f_{L-1}(y_{L-1}))$$
$$\Rightarrow \arg\max_{y_1}(\pi_{y_1}) \arg\max_{y_2}(a_{y_1 y_2}) \arg\max_{y_3}(a_{y_2 y_3})...$$
$$\arg\max_{y_{L-2}}(a_{y_{L-3} y_{L-2}} f_{L-2}(y_{L-2}))$$
$$\Rightarrow \arg\max_{y_1}(\pi_{y_1}) \arg\max_{y_2}(a_{y_1 y_2}) \arg\max_{y_3}(a_{y_2 y_3} f_3(y_3))$$
$$\Rightarrow \arg\max_{y_1}(\pi_{y_1}) \arg\max_{y_2}(a_{y_1 y_2} f_2(y_2))$$
$$\Rightarrow \arg\max_{y_1}(\pi_{y_1} f_1(y_1))$$

In essence, we maximize each state y_i one by one with the help of an auxiliary function f_i which factors out the previous state y_{i-1} for all the possible value of i until only a single state y_0 needs to be maximized. It exploits the Markov property in which only two states need to be maximized for each value of i from the end so that linear time complexity can be achieved with respect to the path length L. Backward tracing is then applied to find the most probable state transition path $Y^* = (y_1^*, y_2^*, y_3^*, ..., y_L^*)$.

Similarly, N-Max-Product algorithm works in the same principle but reserves N paths during the maximization procedure. In other words, we can replace each argument maximization operator '$\arg\max_{y_i}$' by a similar operator which can maximize and store the top N arguments during the dynamic programming shown above.

MOTIF MATRIX DISTANCE

Given two motif matrix A and B of sizes $5\times n_A$ and $5\times n_B$ respectively, we can align them using a profile alignment method (e.g. Needleman-Wunsch global alignment (64). Once we have aligned A and B, we calculate the aligned distance $d_a(A,B)$:

$$d_a(A,B)=\sum_{i=1}^{n_A}\sum_{j=1}^{n_B}\delta(i,j)d(\vec{a_i},\vec{b_j})$$

where $\delta(i,j)=1$ when the ith column of A is aligned to the jth column of B. Otherwise, $\delta(i,j)=0$. $\vec{a_i}$ and $\vec{b_j}$ denote the ith column of A and the jth column of B respectively. $d(\vec{a_i},\vec{b_j})$ is the Euclidean distance between $\vec{a_i}$ and $\vec{b_j}$.

In addition, we also take into account the gap distance $d_g(A,B)$:

$$d_g(A,B)=d_{max}*(min(n_A,n_B)-(\sum_{i=1}^{n_A}\sum_{j=1}^{n_B}\delta(i,j)))$$

where d_{max} is the maximal possible value for the function $d_a(A,B)$ here in the context of motif matrices. Thus we aim at penalizing each unaligned column by a constant d_{max} where the number of unaligned columns is calculated by $(min(n_A,n_B)-(\sum_{i=1}^{n_A}\sum_{j=1}^{n_B}\delta(i,j)))$.

Finally, we combine and normalize the two distances into a single distance measure $d(A,B)$ as follows:

$$d(A,B)=\frac{d_a(A,B)+d_g(A,B)}{min(n_A,n_B)}$$

For DNA motif matrices A and B, it may be desirable to consider its reverse complements as well. Thus we proposed a unified distance measure $D(A,B)$ as follows:

$$D(A,B)=min(d(A,B),d(A_{rc},B),d(A,B_{rc}),d(A_{rc},B_{rc}))$$

where A_{rc} and B_{rc} are the reverse complement motif matrices of the motif matrices A and B respectively.
